# The Postbiotic Activity of *Lactobacillus paracasei* 28.4 Against *Candida auris*

**DOI:** 10.3389/fcimb.2020.00397

**Published:** 2020-08-04

**Authors:** Rodnei Dennis Rossoni, Patrícia Pimentel de Barros, Iatã do Carmo Mendonça, Rebeca Previate Medina, Dulce Helena Siqueira Silva, Beth Burgwyn Fuchs, Juliana Campos Junqueira, Eleftherios Mylonakis

**Affiliations:** ^1^Department of Biosciences and Oral Diagnosis, Institute of Science and Technology, São Paulo State University/UNESP, São José dos Campos, Brazil; ^2^Division of Infectious Diseases, Rhode Island Hospital, Warren Alpert Medical School at Brown University, Providence, RI, United States; ^3^Department of Organic Chemistry, Center for Bioassays, Biosynthesis and Ecophysiology of Natural Products, Institute of Chemistry, São Paulo State University, UNESP, Araraquara, Brazil

**Keywords:** probiotic, postbiotic, *Lactobacillus*, *Candida auris*, biofilms

## Abstract

*Candida auris* has emerged as a medically important pathogen with considerable resistance to antifungal agents. The ability to produce biofilms is an important pathogenicity feature of this species that aids escape of host immune responses and antimicrobial agents. The objective of this study was to verify antifungal action using *in vitro* and *in vivo* models of the *Lactobacillus paracasei* 28.4 probiotic cells and postbiotic activity of crude extract (LPCE) and fraction 1 (LPF1), derived from *L. paracasei* 28.4 supernatant. Both live cells and cells free supernatant of *L. paracasei* 28.4 inhibited *C. auris* suggesting probiotic and postbiotic effects. The minimum inhibitory concentration (MIC) for LPCE was 15 mg/mL and ranges from 3.75 to 7.5 mg/mL for LPF1. Killing kinetics determined that after 24 h treatment with LPCE or LPF1 there was a complete reduction of viable *C. auris* cells compared to fluconazole, which decreased the initial inoculum by 1-logCFU during the same time period. LPCE and LPF1 significantly reduced the biomass (*p* = 0.0001) and the metabolic activity (*p* = 0.0001) of *C. auris* biofilm. There was also a total reduction (~10^8^ CFU/mL) in viability of persister *C. auris* cells after treatment with postbiotic elements (*p* < 0.0001). In an *in vivo* study, injection of LPCE and LPF1 into *G. mellonella* larvae infected with *C. auris* prolonged survival of these insects compared to a control group (*p* < 0.05) and elicited immune responses by increasing the number of circulating hemocytes and gene expression of antimicrobial peptide galiomicin. We concluded that the *L. paracasei* 28.4 cells and postbiotic elements (LPCE and LPF1) have antifungal activity against planktonic cells, biofilms, and persister cells of *C. auris*. Postbiotic supplementation derived from *L. paracasei* 28.4 protected *G. mellonella* infected with *C. auris* and enhanced its immune status indicating a dual function in modulating a host immune response.

## Introduction

Opportunistic infections are caused by non-pathogenic microorganisms which become pathogenic when the body's defense system is impaired (Riccardi et al., 2019). *Candida* spp. can cause vaginitis, oral candidiasis, cutaneous candidiasis, and candidemia. This genus are responsible the main opportunistic yeast infection in the world (Jacobsen et al., 2012; Wachtler et al., 2012; Martins et al., 2014).

Most of these infections originate from biofilms (Vicariotto et al., 2012; Matsubara et al., 2016a,b), defined as complex and dynamic structures consisting of cells encased in a matrix of extracellular polymeric substances (Serra et al., 2015; Rodrigues M. E. et al., 2019). The adhered cells of the biofilm are protected from the external environment by the extracellular matrix that shields them and contributes to the increased antimicrobial resistance. The development of *Candida* spp. biofilms is one of the main reasons that fungal cells exhibit resistance to antifungals (Davies, 2003; Rodrigues et al., 2016; Cernakova et al., 2019; Rodrigues M. E. et al., 2019). Thus, treatment of infections derived from biofilms represents an important challenge today.

*C. auris* is a pathogenic yeast associated with invasive infections and it has been reported from 32 countries across six continents within a decade (Chakrabarti and Singh, 2020). Previous studies suggest that this species is highly tolerant to thermal and osmotic stresses, possessing the ability to persistently colonize hospital environments, and thereby causing outbreaks (Rossato and Colombo, 2018; Spivak and Hanson, 2018; Sabino et al., 2020). Recently, the Centers for Disease Control and Prevention (CDC) published a notice to different public health officials informing them of *C. auris* isolation in U.S. patients (Centers for Disease Control and Prevention, 2016; Sekyere and Asante, 2018). The emergence of *C. auris* is concerning because this yeast has or may develop resistance to different antifungal agents (Lockhart et al., 2017; Chaabane et al., 2019), making infections difficult to treat. Lockhart et al. (2017) evaluated the antifungal susceptibility of 41 isolates of *C. auris* from 54 patients during 2012–2015. The authors found that 93 and 35% of *C. auris* isolates were resistant to fluconazole (FLC) and amphotericin B, respectively. In addition, difficulties in its identification in the routine diagnostic laboratory have a significant impact on outbreak detection and appropriate management (Bidaud et al., 2018).

With poor response to standard antimicrobials and the deficiency in developing new antifungal agents into the clinic, it stands that alternate means of treating the fungal diseases are warranted. In this context, the use of probiotics has been considered a promising strategy for prevention and control of several fungal infections (Mailander-Sanchez et al., 2012; Matsubara et al., 2016a; Hu et al., 2017; Rodrigues C. F. et al., 2019). Probiotics are live microorganisms or microbial cell components that beneficially affect host health (Guarner et al., 2012; Janczarek et al., 2016; Matsubara et al., 2016b; Rossoni et al., 2018), but their clinical application in immunocompromised patients is limited due to the possibility of bacteremia caused by bacteria of the genus *Lactobacillus* (Cannon et al., 2005; Salminen et al., 2006).

To address the risk for live cell probiotics potential of bacteremia, postbiotics have emerged based on the concept that bacterial viability is not essential for probiotics to exert beneficial effects on human health (Tsilingiri and Rescigno, 2013; Cicenia et al., 2014). Postbiotics are products of probiotic bacteria that have biological activity in the host (Mosca et al., 2019) and include metabolites, fractions of microbial cells, fatty acids, proteins, polysaccharides, cell lysates, peptidoglycan derived peptides, and structures responsible for adhesion, such as pili (Wegh et al., 2019). In previous studies by our research group, we isolated the *Lactobacillus paracasei* strain 28.4 from the oral cavity and showed its inhibitory activity against the commensal fungus *C. albicans* in both *in vitro* and *in vivo* models (Rossoni et al., 2017, 2018; de Barros et al., 2018; Santos et al., 2019).

In the present study, the supernatant of *L. paracasei* 28.4 was extracted and fractionated to determine if postbiotic elements could be effective at inhibiting the emergent species *C. auris*. Particularly, examining their effects on biofilm state that can plague immunocompromised patients that spur recurrent and invasive infections, also, we examined cell-free postbiotic supernatant inhibitory activity using the invertebrate infection model *Galleria mellonella* to look for host responses.

## Materials and Methods

### Strains

A panel of *C. auris* strains (designated AR-BANK#0381 to AR-BANK#0390) obtained from the antimicrobial resistance bank of the CDC was used in this study. The strains in this panel are listed in Table 1. *L*. *paracasei* 28.4, an isolate from the oral cavity of a caries-free individual, was used to obtain crude extract and supernatant fractions. Our research group isolated, identified, and characterized this probiotic strain demonstrating its antifungal action both *in vitro* and *in vivo* studies (Rossoni et al., 2017, 2018; de Barros et al., 2018; Santos et al., 2019; Ribeiro et al., 2020).

**Table 1:** *C. auris* isolates used in this study.

**Strain no**.	***C. auris* strain designation**
CAU-01	AR-BANK#0381
CAU-02	AR-BANK#0382
CAU-03	AR-BANK#0383
CAU-04	AR-BANK#0384
CAU-05	AR-BANK#0385
CAU-06	AR-BANK#0386
CAU-07	AR-BANK#0387
CAU-08	AR-BANK#0388
CAU-09	AR-BANK#0389
CAU-10	AR-BANK#0390

All *C. auris* isolates were cultured in 1% yeast extract, 2% peptone, 2% dextrose (YPD broth, Difco, Detroit, USA) for 24 h at 37°C, and *L. paracasei* 28.4 was grown on Man Rogosa and Sharpe (MRS) agar (Difco, Franklin Lakes, NJ, USA) for 48 h at 37°C (5% CO_2_).

### *In vitro* Coinfection Cultures in a Planktonic Environment

Coinfection cultures were performed in 2 mL of BHI broth at 37°C in a rollerdrum according Peleg et al. (2008) with modifications. Standardized inoculants of *C. auris* (1 × 10^5^ cells/mL) and *L. paracasei* 28.4 (1 × 10^8^ cells/mL) were incubated together for 24, 48, and 72 h. At the indicated time points, the CFU were enumerated for each group. YPD plates containing kanamycin (45 μg/mL) and MRS plates containing fluconazole (32 μg/mL) were used to determine *C. auris* and *L. paracasei* CFUs, respectively. Results were obtained from three independent experiments.

### *L. paracasei* 28.4 Postbiotic Elements: Supernatant Preparation, Extraction, and Fractionation

First, the *L. paracasei* 28.4 supernatant was produced according to Ribeiro et al. (2017). An inoculum of 1 mL of the standard suspension (10^7^ cells/mL) was seeded into 6 mL of MRS broth and incubated at 37°C for 24 h under microaerophilic conditions. After this incubation, the broth was centrifuged (5,000 RPM for 10 min) and filtered with a 0.22 μm pore size membrane (MFS, Dublin, CA, USA).

Next, postbiotic elements were obtained according to Medina et al. (2019). In total, 4 L of supernatant were extracted with EtOAc (3 × 50% of each medium volume) for the extraction of the active compounds. After evaporation of EtOAc using a rotary evaporator (Buchi rotavapor, Buchi, São Paulo, Brazil), *L*. *paracasei* 28.4 crude extract (LPCE) was obtained (1.18 g). LPCE crude extract was analyzed using HPLC with two LC6AD pumps, CBM-20A communicator, SIL-10AF automatic injector, and SPD-M20A diode array detector (Shimadzu, Columbia, MD, USA), and the column used was a Luna Phenomenex octadecyl silane (C-18) analytical 250 × 4.6 mm, and gradient elution H_2_O /MeOH (95:05 → 0:100) for 45 min. LPCE was fractionated using 20 g of C18 silica in an open column in which methanol and water were employed as a stationary phase in a polarity gradient (26:74, 51:49, 75:25, and 90:10). Fraction 1 of the supernatant (LPF1) was used in all subsequent assays as well as the LPCE. An aliquot of LPCE and LPF1 were cultured in brain heart infusion (BHI) broth (Sigma, St. Louis, MO, USA) to ensure that there was no microbial growth in the postbiotic elements.

### Minimum Inhibitory Concentration

To determine the minimal inhibitory concentration (MIC) of LPCE and LPF1 against the *C. auris* strains, colonies of each strain were inoculated in 5 mL of yeast peptone dextrose (YPD) media (Sigma, St. Louis, MO, USA) and grown overnight at 37°C. The cells were harvested by centrifugation at 5,000 rpm for 5 min and washed with phosphate-buffered saline (PBS). Subsequently, the cell pellets were suspended in RPMI 1640 medium (Sigma, St. Louis, MO, USA). The cell count was determined using a hemocytometer and adjusted to 1.0 × 10^3^ cells/mL. Susceptibility patterns of the isolates to LPCE and LPF1 were determined by performing the broth microdilution assay. The final concentrations of LPCE and LPF1 ranged from 30 to 0.029 mg/mL. Fluconazole and amphotericin B (Sigma-Aldrich, St. Louis, MO, USA) were used as a positive control and the assay was performed according the Clinical and Laboratory Standards Institute (CLSI) document M27-A2 (National Committee for Clinical Laboratory Standards, 2002). The final concentrations of fluconazole and amphotericin B ranged from 64 to 0.125 μg/mL. The resistance breakpoints were used as described in the Clinical and Laboratory Standards Institute (CLSI) guidelines based on *C. albicans* interpretive breakpoints (Fluconazole: ≤ 8.0 μg /mL for susceptible, ≥64 μg /mL for resistant; Amphotericin B: >1 μg/ml for resistant) (National Committee for Clinical Laboratory Standards, 2002).

### Minimum Fungicidal Concentration

The minimum fungicidal concentration (MFC) was determined as follows. In total, 10 μL of yeast culture from each microwell of the MIC assay was plated on YPD agar and incubated at 35°C overnight. The static/cidal parameter can be roughly estimated by comparing the MFC of a given antifungal to its MIC. If the MFC is ≤ 4 × MIC the drug is considered cidal (Pfaller et al., 2004).

### Time to Kill Assays

After the MIC test of all *C. auris* strains, the CAU-01 strain was selected for the subsequent tests based on its sensitivity to fluconazole and amphotericin B. This strain showed the lowest MIC value for both antifungals, a requirement for inducing persister cells. Therefore, *C*. *auris* strain CAU-01 was explored to interrogate the killing effects of LPCE and LPF1. The assays were carried out in 10-mL tubes (BD Biosciences, San Diego, CA, USA) in triplicate according to Tharmalingam et al. (2019) with modifications. Briefly, log-phase cultures of CAU-01 were diluted in fresh RPMI medium to a density of 10^6^ cells/mL LPCE and LPF1 were added at concentrations 3.75–120 mg/mL (corresponding to 1 × MIC−8 × MIC), and the tubes were incubated at 37°C with agitation (200 rpm). Portions of cell suspensions were withdrawn at predetermined time points (24, 48, and 72 h). Serial dilutions were plated on YPD agar to determine the colony-forming unit/mL (CFU/mL) of the cell suspensions. CFU were determined after incubation for 48 h at 37°C. Three independent experiments were performed. As a positive control, we included the antifungal agent fluconazole at 4, 16 and 32 μg/mL (corresponding to 1 × MIC−8 × MIC).

### Biofilm Formation

Evaluation of biofilm formation was performed in 96-well microtiter plates (Corning, New York, NY, USA) following the methodology described by Vilela et al. (2015) and Rossoni et al. (2018), with some modifications. Briefly, 100 μL of *C. auris* standard suspensions (1.0 × 10^7^ cells) were deposited into 96-well microtiter plates, after which the plates were placed on a 75-rpm shaking incubator at 37°C for 90 min. Each well was washed twice with PBS, and 200 μL of yeast nitrogen base (YNB) broth (Sigma, St. Louis, MO, USA) supplemented with 100 mM glucose with LPCE or LPF1 were added to the wells of each plate at the concentrations of 0.5 × MIC, 1 × MIC, and 2 × MIC. For the control groups, 200 μL of YNB broth supplemented with 100 mM glucose without LPCE or LPF1 was added. The plate was incubated for 48 h at 37°C with shaking at 75 rpm. The liquid medium was replaced after 24 h and the treated groups received fresh LPCE, LPF1, or fluconazole dilutions. After the incubation period, each well was washed twice with PBS for subsequent analysis of total biomass and metabolic activity. As a positive control, we included the antifungal agent fluconazole at 4 and 8 μg/mL (corresponding to 1 × MIC and 2 × MIC).

### Analysis of Biofilms by Total Biomass Quantification

After biofilm formation, the biofilm biomass was quantified utilizing an assay previously described by Peeters et al. (2008), with modifications. For biofilm fixation, 100 μL of 99% methanol was added to the wells (Sigma-Aldrich, St. Louis, MO, USA). After 15 min, the supernatants were removed and the plates were air-dried. Then, 100 μL of a 1% crystal violet (CV) solution was added to all wells. After 20 min, the residual CV solution was removed by washing with PBS. Finally, bound CV was released by adding 150 μL of 33% acetic acid (Sigma-Aldrich, St. Louis, MO, USA). The absorbance was measured at 540 nm. All steps were carried out at room temperature. The CV assay was performed as two independent experiments with *n* = 6 biofilms per group.

### Analysis of Biofilms by XTT Reduction Assay Colorimetric Assay

The biofilms formed also were evaluated by a metabolic assay based on the reduction of XTT, a tetrazolium salt (Sigma-Aldrich, St. Louis, MO, USA) according Jin et al. (2004) and Rossoni et al. (2019). Briefly, XTT salt was dissolved in PBS at a final concentration of 1 mg/mL. Immediately before each assay, a menadione (Sigma-Aldrich, St. Louis, MO, USA) solution was prepared at a final concentration of 0.4 mM and filter-sterilized. The XTT solution was thawed prior to each assay and mixed with the menadione solution at a ratio of 20:1 (v/v). Each well was washed two times with 200 μL of PBS to remove any non-adherent cells. Next, 158 μL of PBS, 40 μL of XTT, and 2 μL of menadione were added to each of the pre-washed wells. The plates were incubated in the dark at 37°C for 3 h. Afterwards, 100 μL of the solution was transferred to a new well, and any colorimetric change in the solution was measured using a microtiter plate reader (Tp Reader; Thermo Plate, Shenzhen, China) at 490 nm. The XTT assay was performed as two independent experiments with *n* = 6 biofilms per group.

### Isolation and Susceptibility of *C. auris* Persisters Cells

For this study, the methodologies described by LaFleur et al. (2006) and Al-Dhaheri and Douglas (2008) were used with some modifications. Briefly, *C. auris* was grown for 72 h at 37°C with shaking in RPMI 1640 with l-glutamine and 0.165 M MOPS growth medium (50 mL in 250-ml flasks) to isolate persister cells in a stationary-phase cultures. After 72 h, cells from the stationary-phase cultures were harvested and washed twice in PBS. All cell suspensions were adjusted to concentrations of ~5 × 10^8^ cells/mL. An aliquot of 1 mL of this suspension, containing 10x, 50x, 100x, 150x, and 200x MICs of indicated antifungals (amphotericin B and fluconazole), was added to the wells of a 2 mL deep well assay block (Corning Costar 3960) and incubated at 37°C, with shaking at 225 rpm for 48 h. In groups containing postbiotic elements, the stationary-phase culture suspension was treated with 10 × MIC of LPCE or LPF1. Control cells were treated similarly with buffered medium without antifungal agent. At designated times, 50 μL samples were removed, serially diluted, and spot-plated on YPD agar plates to enumerate the number of persister cells. This experiment was conducted in triplicate.

### *G. mellonella* Survival

*G. mellonella* survival was evaluated following the methodologies described by Mylonakis et al. (2005) and Rossoni et al. (2017), with some modifications. *G. mellonella* (Vanderhorst Wholesale, St. Marys, OH, USA) in their final larval stage were stored in the dark and used within 7 days from shipment. Sixteen randomly chosen *G. mellonella* larvae with similar weight and size (250–350 mg) were used per group in all assays. Two control groups were included in the assays—one group was inoculated with phosphate-buffered saline (PBS), while the other group received no injection as a control to evaluate general viability.

In view of the lack of studies in the literature that used postbiotics elements on *G. mellonella* as an experimental model, the toxicity of LPCE and LPF1 was evaluated prior to the study of experimental candidiasis. The postbiotic elements were injected directly into the hemolymph of *G. mellonella* at varying concentrations (10–80 mg/kg), and survival curves were constructed. Larvae were incubated at 37°C and monitored daily for survival.

For the experimental candidiasis, *C. auris* suspension was prepared from cultures in 5 mL of YPD liquid medium and incubated at 37°C for 18 h. Afterwards, cells were centrifuged at 2,000 × *g* for 10 min, and the supernatant was discarded. The cell pellet was washed twice and suspended in PBS. Cell densities were adjusted using a hemocytometer.

To evaluate the effects of LPCE or LPF1 on *C. auris* infection, the larvae were pre-treated by injecting the LPCE (80 mg/kg) or LPF1 (30 mg/kg) through the last left proleg (volume, 10 μL). After 2 h, larvae were infected with 10^6^ cells/larvae of *C. auris* suspended in PBS at the last right proleg (10 μL of volume). Larvae were incubated at 37°C and monitored daily for survival. Larvae were considered dead when they displayed no movement in response to touch.

### Quantification of *G. mellonella* Hemocytes

In order to investigate the immunological mechanisms associated with the postbiotics elements against infection by *C. auris*, larvae were pre-treated with LPCE (80 mg/kg) or LPF1 (30 mg/kg) and infected with *C. auris* as described above. Hemocytes were collected from the hemocoel at 4 h post-injection with *C. auris*. Larvae were bled into tubes containing cold, sterile insect physiologic saline (IPS) (150 mM sodium chloride; 5 mM potassium chloride; 100 mM Tris—hydrochloride, pH 6.9 with 10 mM EDTA, and 30 mM sodium citrate). The hemocytes were identified based on cell morphology and quantified using a hemocytometer. The results were averaged from four replicates.

### Analysis of Peptide Expression

*G. mellonella* gene expression was evaluated following the methodologies described by Mowlds et al. (2010) and Rossoni et al. (2017). After pre-treatments and infection, larval RNA was extracted using TRIzol (Ambion, Inc., Carlsbad, CA, USA) at 24 h post-injection of LPCE or LPF1. In brief, a 2 mL volume of TRIzol was added to a 15 mL tube containing the homogenized frozen tissue of larvae and incubated at room temperature (RT) for 10 min. Subsequently, 400 μL of chloroform (Sigma-Aldrich, St. Louis, MO, USA) was added and the tubes were centrifuged at 12,000 × *g* for 15 min at 4°C. The supernatant was then transferred to a new tube, and 1 mL of isopropanol (Sigma-Aldrich, St. Louis, MO, USA) was added. After centrifugation, the obtained pellet was washed with 70% ethanol (Sigma-Aldrich, St. Louis, MO, USA), centrifuged again, and suspended in 50 μL of nuclease-free water (Ambion Inc., Carlsbad, CA, USA). The concentration, purity and quality of the RNA were verified using a NanoVue Plus spectrophotometer (GE Healthcare Bio-Sciences, Pittsburgh, PA, USA).

The extracted total RNA (1 μg) was transcribed into complementary DNA (cDNA) using the Verso cDNA Synthesis Kit (Thermo Fisher Scientific Inc, Waltham, MA, USA), according to the protocols recommended by the manufacturer. The primers for the genes that encode β-actin and galiomicin were designed by Rossoni et al. (2017) and described in Table 2. The transcribed cDNAs were amplified for relative quantification of the expression of the gene encoding galiomicin in relation to the concentration of the reference gene (β-actin).

**Table 2:** List and description of genes and primers sequences used in the qPCR.

**Gene name**	**NCBI genebank**	**Primer sequence (5^**′**^–3^**′**^)**	Product size (bp*)	**Primers source**
Galiomicin	AY528421.1	^*a*^**F**-TCCAGTCCGTTTTGTTGTTG	123 bp	Rossoni et al., 2017
		^*b*^**R**-CAGAGGTGTAATTCGTCGCA		
β-actin	XM_026909080.1	^*a*^**F**- ACAGAGCGTGGCTACTCGTT	104 bp	Rossoni et al., 2017
		^*b*^**R**- GCCATCTCCTGCTCAAAGTC		

a *F indicates a forward primer*.

b *R indicates a reverse primer*.

* *Base pair*.

### Statistical Analysis

The Student's *t*-test was used to compare the results from the cell-cell interaction, time to kill assay, and *in vitro* biofilm assay. Percent survival and killing curves of *G. mellonella* were plotted and statistical analysis was performed by the Kaplan-Meier test using Stata Statistical Software (Stata Corp LP, College Station, TX, USA). Analysis of variance (ANOVA) and Tukey post-test were used to compare the results obtained in the data of hemocyte count and in the analysis of gene expression. All the tests were performed using GraphPad Prism statistical software (GraphPad Software, Inc., California, CA, USA) and a *P*-value ≤ 0.05 was considered significant.

## Results

### Probiotic Effect

**Figure 1:**
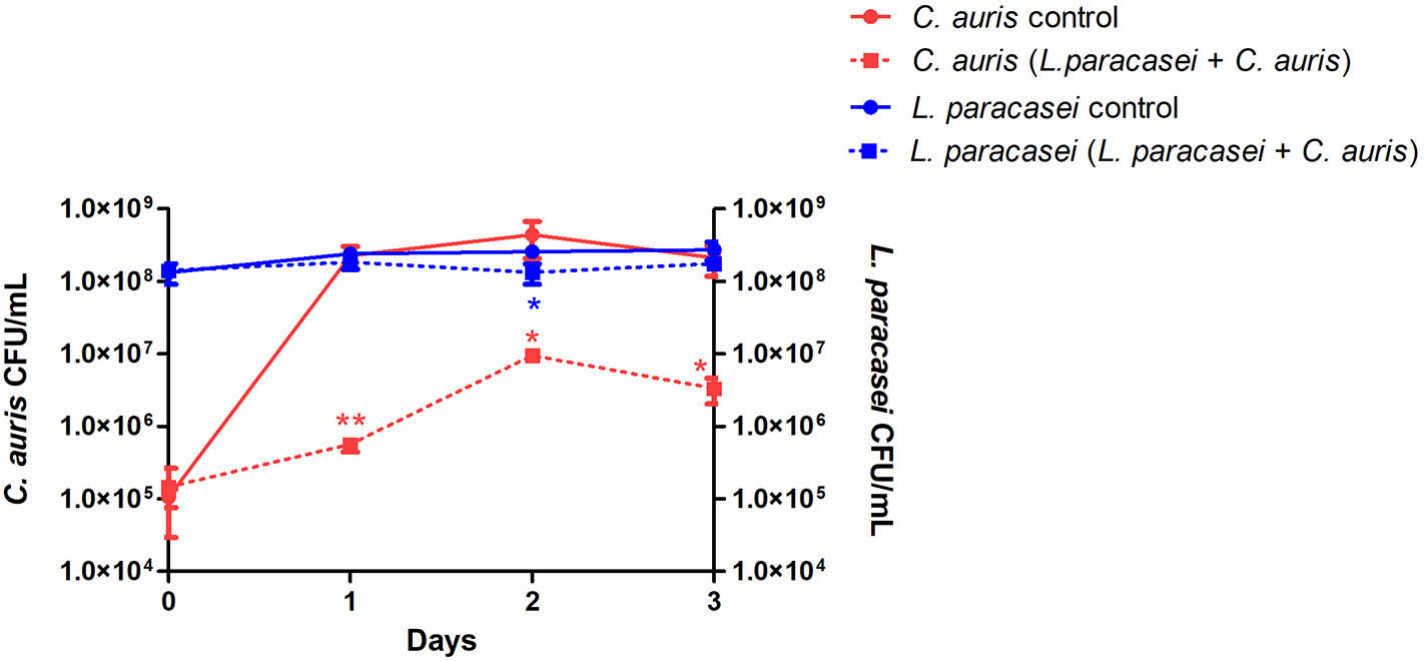
The viability of *C. auris* CAU-01 (solid line) was significantly reduced when co-cultured with *L. paracasei* 28.4 (dashed line). Student *t*-test, *p ≤ 0.01, **p ≤ 0.001.

First, *L. paracasei* 28.4 was screened for antifungal activity against *C. auris* CAU-01 using co-culture. For this purpose, *L. paracasei* 28.4 cells were co-cultured with *C. auris* for 1–3 days. There was a significant reduction in *C. auris* counts for all evaluated times (1 day: 3.6 Log, 2 days: 1.67 Log, and 3 days: 1.8 Log) compared to the control group as demonstrated in Figure 1. *L. paracasei* concentration was constant throughout the assay, however *C. auris* experienced growth suppression in the presence of *L*. *paracasei* 28.4. This probiotic effect can indicate a better ability of *L*. *paracasei* to utilize nutrients in the media for growth, a direct cell-cell interaction, or possibly inhibition by bacterial metabolites.

### Postbiotic Planktonic Inhibition

**Figure 2:**
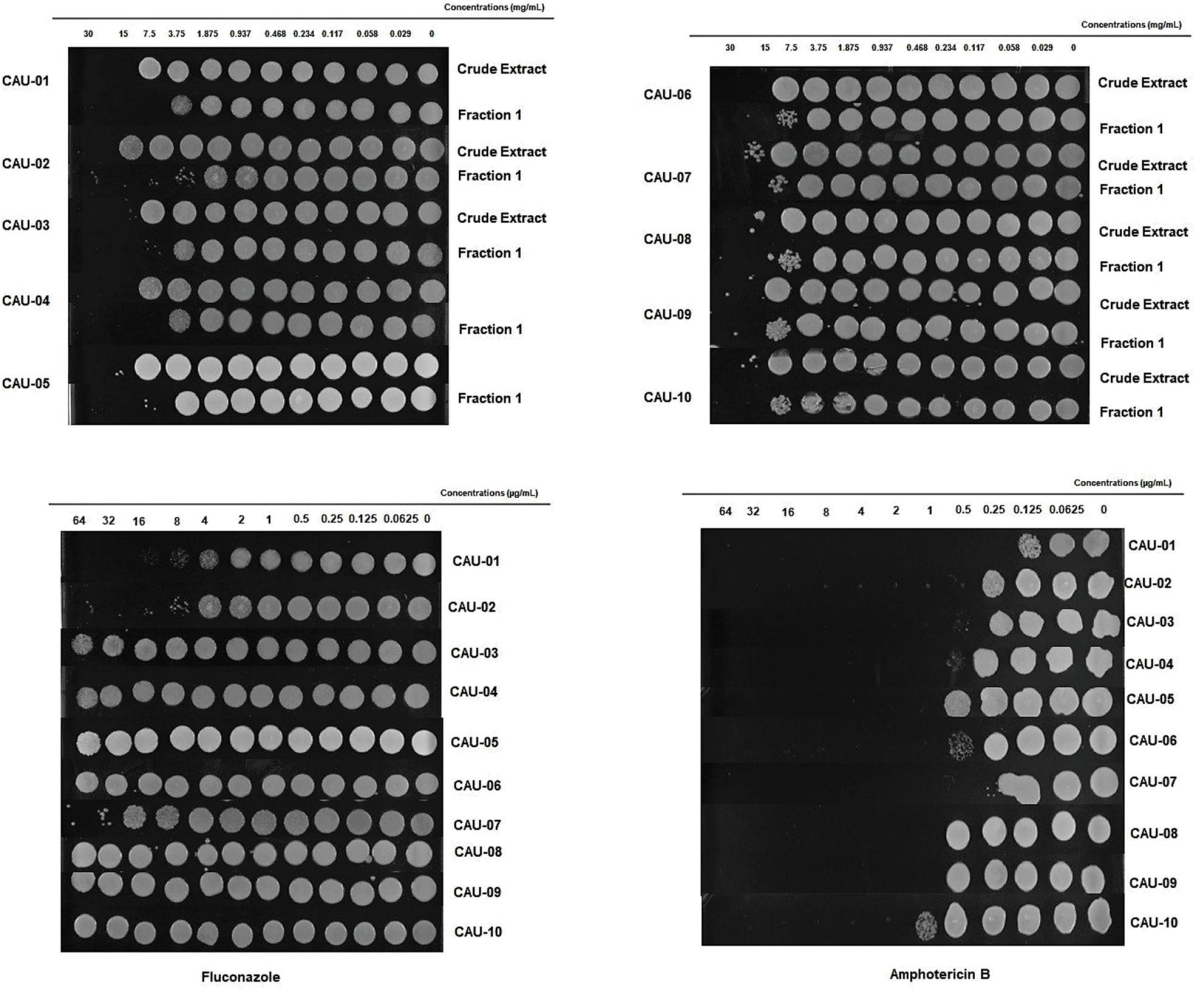
LPCE and LPF1 inhibits *C. auris*. The static vs. cidal nature of the inhibition was examined for the various postbiotics concentrations. Growth at concentration equivalent or higher than the MIC indicated that LPCE and LPF1 were fungistatic and inhibition of growth indicated fungicidal activity.

To explore the possibility of an indirect inhibitory activity posed by bacterial metabolites, we investigated *C*. *auris* inhibition by cell-free supernatant of *L. paracasei* 28.4. Inhibitory assessment was made using crude extract (LPCE) and a fraction (LPF1) derived from the *L. paracasei* supernatant. The MIC of LPCE and LPF1 was evaluated for 10 strains of *C. auris* (Table 3). MICs for LPCE were 15 mg/mL for all strains, and LPF1 MICs ranged from 3.75 to 7.5 mg/mL. For all strains evaluated, the MFC values were ≤ 4 × MIC, so the postbiotic elements were considered cidal (Figure 2). Thus, there does appear to be a postbiotic effect by *L*. *paracasei* 28.4. The lower MIC of LPCE is reasonable considering that the active component is more diluted in the unfractionated volume.

**Table 3:** MIC for *C. auris* strains.

**Specie**	**Strain**	**LPCE**	**LPF1**	**Fluconazole**	**Amphotericin**
		**(mg)**	**(mg)**	**(μg)**	**(μg)**
*C. auris*	CAU-01	15	3.75	8	0.125
*C. auris*	CAU-02	15	3.75	16	0.25
*C. auris*	CAU-03	15	7.5	>64	0.5
*C. auris*	CAU-04	15	3.75	>64	0.5
*C. auris*	CAU-05	15	7.5	>64	0.5
*C. auris*	CAU-06	15	7.5	>64	0.5
*C. auris*	CAU-07	15	7.5	8	0.25
*C. auris*	CAU-08	15	3.75	>64	1
*C. auris*	CAU-09	15	7.5	>64	1
*C. auris*	CAU-10	15	7.5	>64	2

**Figure 3:**
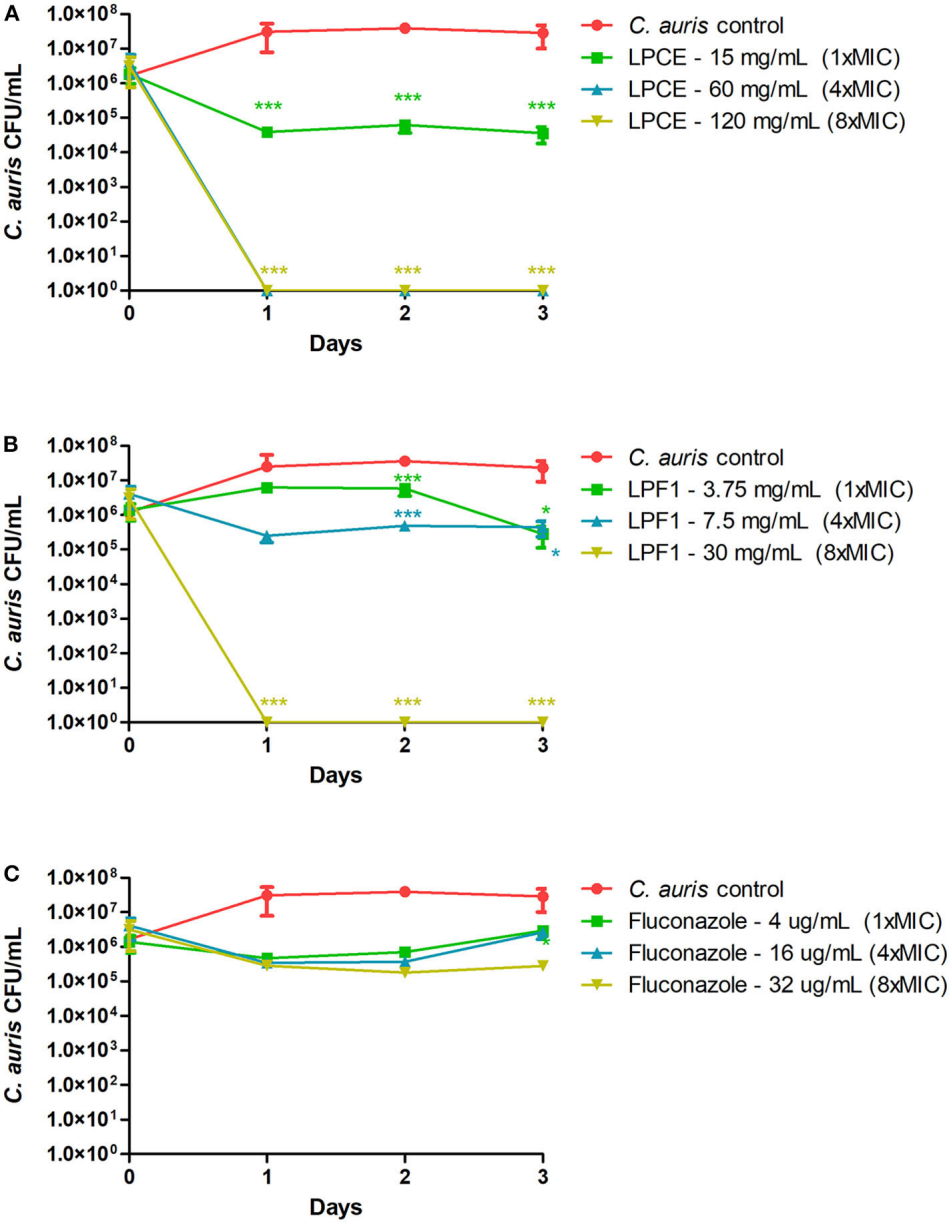
Killing kinetics. **(A)** Growth curves were generated using *C. auris* cells treated with LPCE. **(B)** Growth curves were generated using *C. auris* cells treated with LPF1. **(C)** Growth curves were generated using *C. auris* cells treated with fluconazole. Student *t*-test, *p ≤ 0.01, ***p ≤ 0.0001.

Since LPCE and LPF1 were active against all of the tested *C. auris* strains, we selected a single strain (CAU-01) for follow-up experiments. The killing kinetics assay determined that the total viable fungal count was about 6-log CFU at 0 h. After 24 h, there was a complete reduction of the total viable count of *C. auris* cells treated with LPCE (4 × MIC: 60 mg/mL) or LPF1 (8 × MIC: 30 mg/mL) (Figure 3). As a positive control, we included the antifungal agent fluconazole. Fluconazole decreased the initial inoculum by 1-log CFU during the same time period at all concentrations tested (1 × MIC: 4 μg/mL; 4 × MIC: 16 μg/mL, and 8 × MIC: 32 μg/mL).

### Postbiotic Biofilm Inhibition

**Figure 4:**
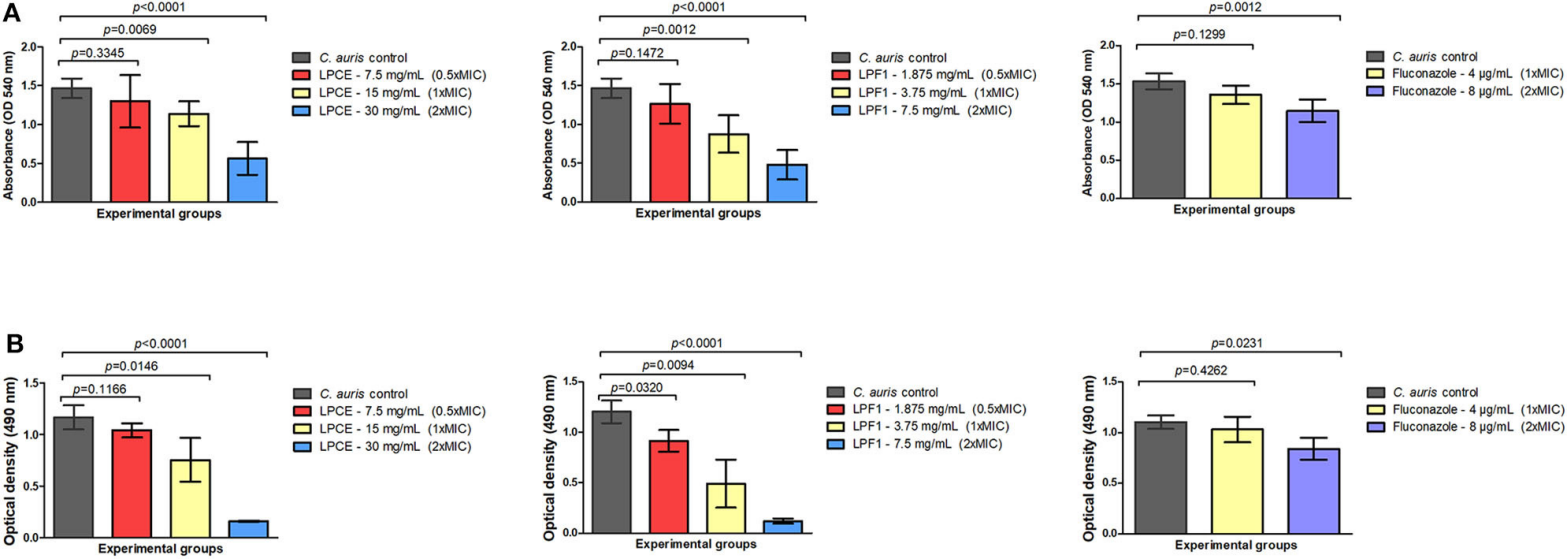
Quantification of cells in biofilms formed at the bottom of 96-well plates. **(A)** Biomass quantification and **(B)** Metabolic activity (XTT analysis) of different treatments (LPCE, LPF1, and Fluconazole). The different concentrations of LPCE and LPF1 used in biofilm formation correspond to 0.5 × MIC, 1 × MIC, and 2 × MIC values. Student's *t*-test, *p* ≤ 0.05.

It is known that antifungal compounds have variable efficacy against biofilms. Therefore, the cell free supernatant extract and fraction were tested for inhibitory activity on biofilms. LPCE and LPF1 at 1 × MIC and 2 × MIC concentrations significantly reduced the biomass (*p* = 0.0001) and the metabolic activity (*p* = 0.0001) of the *C. auris* biofilm as shown in Figures 4A,B, respectively. A biomass of *C. auris* showed a reduction of up to 67% for LPF1, 61% for LPCE, and 21% using fluconazole. The metabolic activity of biofilms reduced 89, 85, and 23% for LPF1, LPCE, and fluconazole, respectively. The biofilms treated with the postbiotic elements in the concentration of 0.5 × MIC also caused a reduction in relation to the control groups but there was no statistically significant difference, indicating a dose dependent effect on the biofilms.

**Figure 5:**
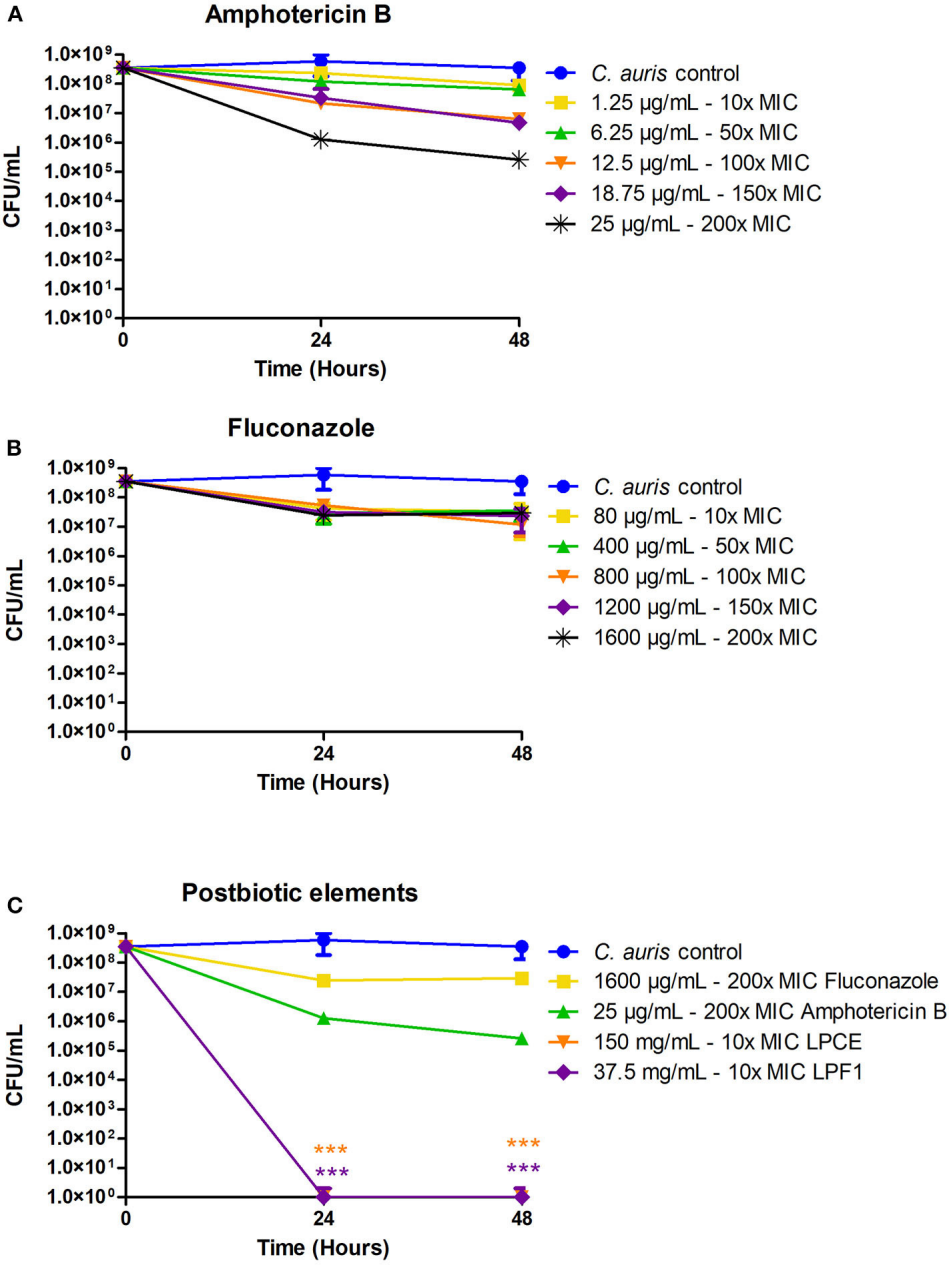
Isolation of *C. auris* persisters. **(A)** *C. auris* persisters cells induced by amphotericin B. **(B)** *C. auris* persisters cells induced by fluconazole. **(C)** LPCE and LPF1 were able to kill all persister cells. Student's *t*-test, ***p ≤ 0.0001.

We sought to determine the susceptibility of persister cells to the *L*. *paracasei* 28.4 supernatant derived elements. To generate *C. auris* persisters, *C. auris* CAU-01 was grown to stationary phase and then was treated with different concentrations of amphotericin and fluconazole (10x, 50x, 100x, 150x, and 200x MIC) or postbiotic elements (10 × MIC) for 48 h. The concentration of cells in stationary phase was 5 × 10^8^ CFU/mL (~8.54 Log) and, after treating with 200 × MIC (25 μg/mL) amphotericin B or 200 × MIC (1,600 μg/mL) fluconazole for 48 h, the cell viability was ~10^4^ CFU/mL and ~10^7^ CFU/mL, respectively. Also, we treated the cells in stationary phase with a dose of 10 × MIC LPCE (150 mg/mL) or LPF1 (37.5 mg/mL) for 48 h and there was a complete reduction in the growth of *C. auris* cells. *C. auris* was tolerant of standard of care medications fluconazole and amphotericin at concentrations that are normally detrimental to the fungi, suggesting a persistent state. Under the same conditions, exposure to LPCE, and LPF elicited a cell reduction, suggesting the ability to inhibit persister cells (Figure 5).

### Postbiotic Treatment in the *G*. *mellonella* Infection Model

**Figure 6:**
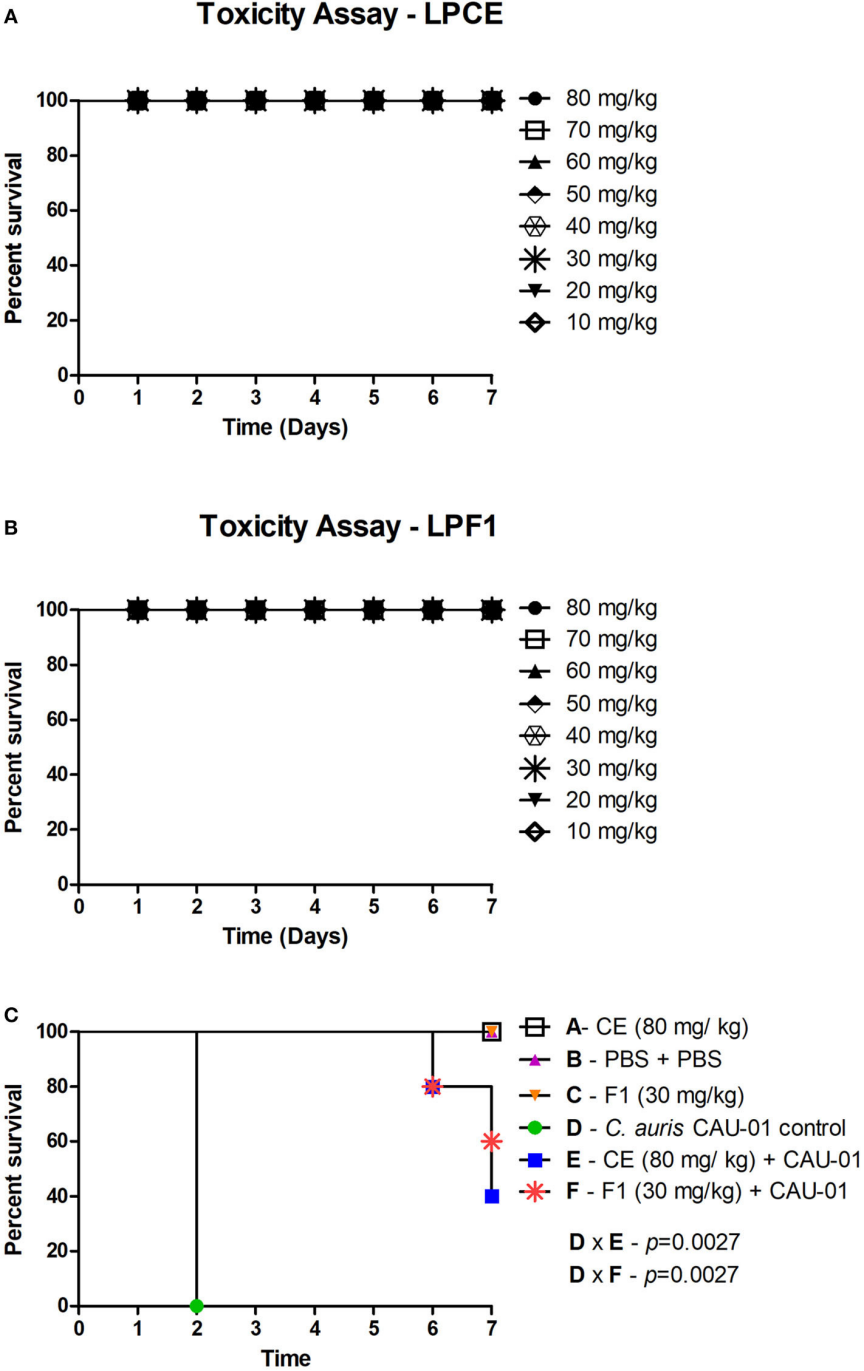
**(A)** Toxicity evaluation of LPCE in *G. mellonella* model. **(B)** Toxicity evaluation of LPF1 in *G. mellonella* model. *G. mellonella* larvae were injected with serial concentrations of postbiotic elements. No death was observed at the concentrations used. **(C)** LPCE and LPF1 prolong the survival of *G. mellonella* larvae infected with *C. auris*. Significant differences were observed in survival between the “LPCE (80 mg/mL) + CAU-01 group” and “PBS + *C. auris* CAU-01 control group” and between the “LPF1 (30 mg/mL) + CAU-01 group” and “PBS + *C. auris* CAU-01 control group.” Kaplan-Meier test, *p* ≤ 0.05.

First, *G. mellonella* was used to evaluate acute systemic toxicity of the postbiotic elements. The larvae were injected with varying concentrations of LPCE and LPF1 (80–10 mg/kg), and their survival was monitored for a period of 7 days. LPCE and LPF1 did not exert toxic effects on the larvae when administered at those concentrations (Figures 6A,B).

To investigate the antifungal effects of LPF1 and LPCE in a *G. mellonella* model, we tested the efficacy of pretreatment with LPF1 and LPCE in larvae infected with *C. auris*. LPF1 and LPCE were injected into the larvae at 2 h prior to infection with *C. auris* concentrations of 30 and 80 mg/kg, respectively. In the control group, infection with *C. auris* without previous injection of postbiotics elements caused death in 100% of the larvae within 3 days. When the larvae were pretreated with LPF1 or LPCE prior to *C. auris* infection, the survival rate of *G. mellonella* larvae was significantly prolonged (*p* < 0.05) (Figure 6C). More specifically, larval survival increased 43% for LPF1 and 37% for LPCE groups.

**Figure 7:**
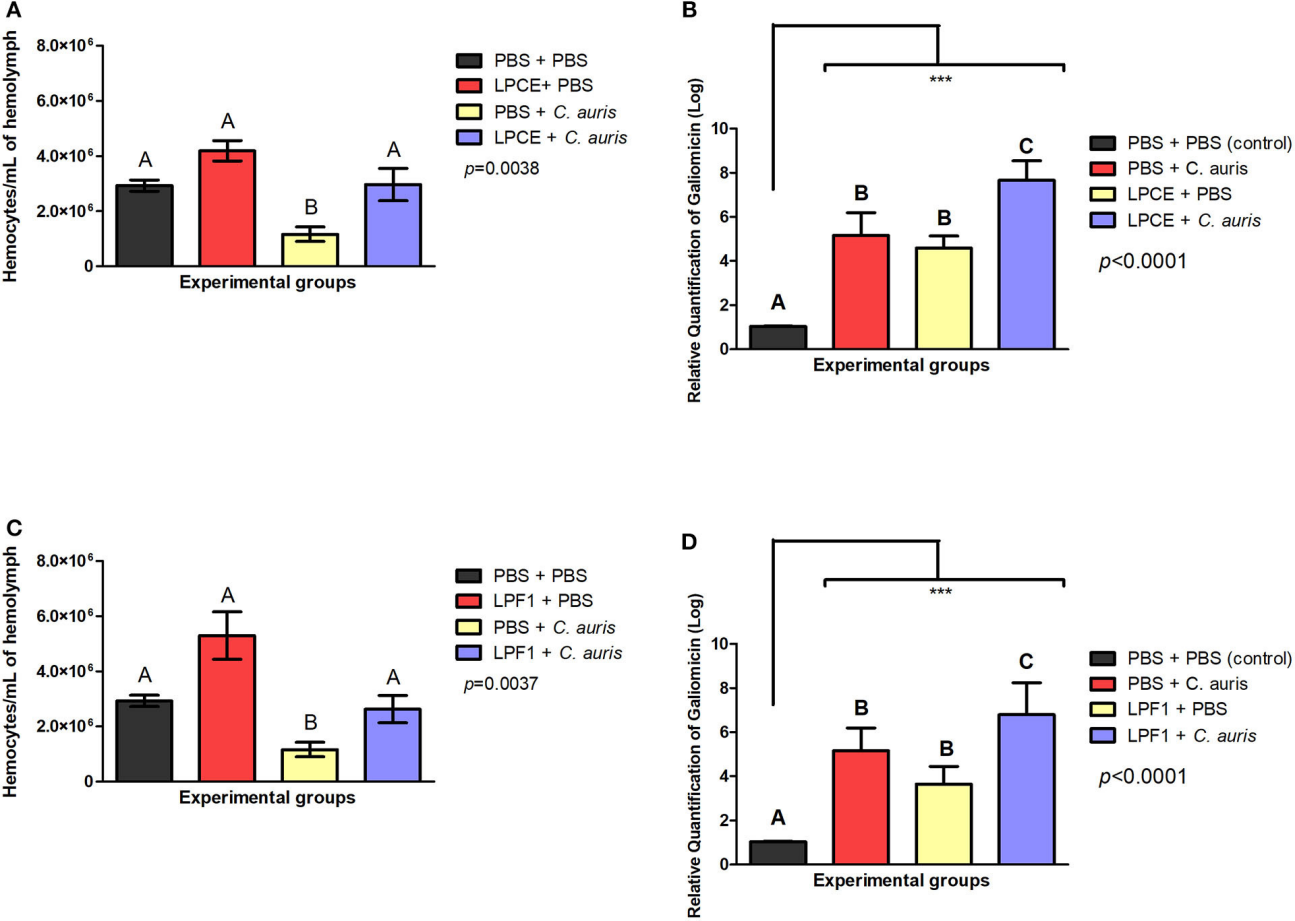
LPCE and LPF1 modulate the immune system of *G. mellonella*. **(A)** LPCE: The group of LPCE + PBS increased the hemocyte number compared to a PBS control (PBS + PBS). The group LPCE + *C. auris* also increased the hemocyte quantity compared to *C. auris* group (PBS + *C. auris*). **(B)** LPF1: The group of LPF1 + PBS increased the hemocyte number compared to a PBS control (PBS + PBS). The group LPF1 + *C. auris* also increased the hemocyte quantity compared to *C. auris* group (PBS + *C. auris*). PBS + *C. auris* group showed a reduction of hemocyte quantity in relation to the PBS control group, but when the larvae were pretreated with LPCE or LPF1, the hemocyte quantity was very similar to the values found in the PBS control group. **(C,D)** LPCE and LPF1 increased the gene expression of galiomicin of *G. mellonella*. Relative quantification (log) of galiomicin for the groups treated with only PBS (Control), pre-treated with PBS and infected with *C. auris*, only treated with LPCE or LPF1, and pre-treated with LPCE or LPF1 and infected with *C. auris*. The units in the Y-axis were calculated based on the 2^−ΔΔCT^ method, and they are expressed as the means and standard deviation. Galiomicin expression was normalized and compared with the expression of insects exposed to the control (PBS) using the reference gene β-actin. Different letters (A, B, and C) represent statistically significant differences among the groups. ANOVA and Tukey Tests (*p* < 0.05 was considered significant). ***p ≤ 0.001.

To investigate the immune mechanisms associated with the effects of LPCE and LPF1 against *C. auris* infection, we determined the number of available hemocytes in the hemolymph of larvae after 4 h of *C*. *auris* injection. Hemocyte count was performed after 4 h of infection based on our previous study in which *L. paracasei* 28.4 stimulated hemocyte production in 4 h rather than 24 h (Rossoni et al., 2017). We analyzed only the larvae not infected by *C. auris* and observed an increase in the number of hemocyte in the LPCE (1.43-fold increase) (Figure 7A) and LPF1 (1.8-fold increase) (Figure 7B) groups compared to the PBS control group, but there was no statistically significant difference. In the larvae infected with *C. auris*, the groups pretreated with LPCE or LPF1 also showed increased hemocyte numbers compared to the *C. auris* control group (*p* < 0.05). The LPCE and LPFI groups reached, respectively, 2.55 (Figure 7A) and 2.26-fold increase (Figure 7B). Interestingly, we also observed that the *C. auris* group showed a reduction of hemocyte numbers in relation to the PBS control group in agreement with Bergin et al. (2003) that demonstrated an inverse relationship with infectious fungi and hemocyte density, but when the larvae were pretreated with LPCE or LPF1, the hemocyte quantity was very similar to the values found in the PBS control group (Figures 7A,B).

The presence of an increased hemocyte count suggests that LPCE and LPF1 could modulate the immune response of *G. mellonella* larvae. Thus, we further explored alterations in the immune response examining the expression of the antifungal peptide galiomicin. For this assay, we evaluated the galiomicin expression after 24 h since the greatest expression of this peptide occurs at a later stage of *Candida* infection (Rossoni et al., 2017). We found that LPCE and LPF1 were able to increase the expression of galiomicin. The groups pretreated with LPCE or LPF1 and then infected with *C. auris* had a statistically significant increase (*p* < 0.0001) in relation to the control group infected by *C. auris* (LPCE group: 1.48-fold increase; LPF1 group: 1.31-fold increase). LPCE and LPF1 induced an increase in gene expression of 4.58 and 3.64-fold compared, respectively, to the control group formed by consecutive PBS injections (Figures 7C,D). These results indicate that LPCE and LPF1 increased the hemocyte density and levels of galiomicin expression, which may protect *G. mellonella* from *C. auris* infection.

## Discussion

Recently, postbiotics have gained more and more attention due to their beneficial actions on the host without the adverse risk of inducing bacteremia in immunocompromised patients from the delivery of live cells (Gao et al., 2019). In this study, we identified LPCE and LPF1 derived from *L. paracasei* 28.4 supernatant as postbiotics and a potentially alternative antifungal treatment against *C. auris*, a globally emerging pathogen. The results obtained in the cell-cell interaction demonstrated that *L. paracasei* strain 28.4 was able to interfere with *C. auris* growth, demonstrating the potential for probiotic activity against *C. auris*. Also, the postbiotic elements reduced *C. auris* in planktonic, biofilm, and persister states, a significant feat. In *in vivo* assays, LPCE and LPF1 protected *G. mellonella* infected with *C. auris*. Our research provides a novel idea for prevention and treatment of *C. auris* infections.

The MIC results of this study demonstrated the antifungal activity of LPCE and LPF1 against all 10 *C. auris* strains, including both fluconazole-sensitive and fluconazole-resistant strains. Although the MIC values found were higher than fluconazole, the postbiotic elements are probably composed of a pool of molecules and are not yet fully purified. In addition, it was observed that the higher its purification and subsequent fractionation, the lower the MIC value obtained (MIC value of LPF1 is four times less than the value of LPCE).

The antibacterial properties of postbiotics have been tested on bacterial diseases; for example, Dunand et al. (2019) determined the protective capacity of postbiotics of fermented milk against *Salmonella enterica* serovar Typhimurium. The authors obtained the postbiotic from five frozen commercial cultures of thermophilic lactobacilli and 11 autochthonous *Lactobacillus* strains. The use of postbiotics for 14 days significantly increased the secretory IgA in feces of mice and a higher survival was also observed compared to controls demonstrating an immunomodulatory and protective capacity against *S. enterica* serovar Typhimurium infection in mice. In addition, studies demonstrate the beneficial role of postbiotics in inflammatory activity (Tsilingiri et al., 2012; Compare et al., 2017; Gao et al., 2019; Johnson et al., 2019). To our knowledge, this is the first study supporting the use of a postbiotic from *Lactobacillus* cells against *C. auris* and its use remains unexplored as a therapeutic option in these fungal infections. Therefore, further studies are needed to evaluate some important aspects such as toxicity, adverse effects, and viability of mass production.

To investigate the antifungal potential of LPCE and LPF1, we evaluated its effects in the growth kinetics of *C. auris*. The use of LPCE and LPF1 completely inhibited *C. auris* growth in contrast to the clinical antifungal agent fluconazole, which did not demonstrate any efficacy more than 1-Log. LPCE and LPF1 also exerted anti-biofilm activity against *C. auris*; significant reductions in biofilm formation were observed in both biomass amount and metabolic activity. In agreement to this result, Rossoni et al. (2018) evaluated the antifungal action of the *L. paracasei* 28.4 supernatant on different *C. albicans* strains. The raw supernatant of *L. paracasei* 28.4 was capable of reducing the growth of *C. albicans* by up to 73% in planktonic cultures, 62% in biofilms and interferes negatively in adhesion (*ALS3*: 66-fold decrease) and hyphae formation genes (*HWP1*: 66-fold decrease; *CPH1*: 1000-fold decrease). Although the postbiotic elements have had effectiveness to reduce *C. auris* biofilms, previous studies have shown that biofilm formation is highly variable between different strains of *C. auris* and this fact merits further exploration (Sherry et al., 2017; Pathirana et al., 2018).

The biofilms of *C. auris* may become increasingly resistant to conventional antifungals according to their formation time. For example, after 4 h of biofilm development, the median MIC increased 16-fold for miconazole and 4-fold for amphotericin B compared to the 0 h time of biofilm formation (Kean et al., 2018). Borman et al. (2016) demonstrated that old cultures of *C. auris* can survive in high concentrations of fluconazole (256 μg/mL), as well as be unresponsive to treatment *in vivo* using *G. mellonella* model. These facts agree with the low sensitivity of biofilms to fluconazole found in this study and reinforce the search for alternative treatments such as postbiotics in *C. auris* infections.

Biofilms harbor drug resistant cells, included among them are persister cells which, in their metabolically dormant state, can be recalcitrant to antifungal agents (LaFleur et al., 2006). One important aspect of postbiotic elements was their ability to eliminate all *C. auris* persister cells that survived high dosages of amphotericin B or fluconazole. Persister cells were reported for the first time as a subpopulation of bacteria tolerant to a particular antibiotic that, after removal of the antimicrobial agent, gave rise to a new population of susceptible microbial cells (Bigger, 1944). In the clinical setting, persisters are usually associated with recurrent infections and the development of chronic infections (Denega et al., 2019). The first report of *Candida* spp. persister cells was described for LaFleur et al. (2006) that showed a biphasic killing curve when *C. albicans* was exposed to amphotericin B, chlorhexidine, or a combination of both. In addition, Al-Dhaheri and Douglas (2008) showed that not all *Candida* spp. and strains are able to form persister cells, for example, *C. albicans* strain SC5314 (Gillum et al., 1984), one of the most commonly used *C. albicans* strains used for molecular genetics studies, is not able to form persister cells *in vitro* (Denega et al., 2019).

The alternative invertebrate model of *G. mellonella* was used to evaluate protective effects of LPCE and LPF1 in experimental candidiasis by *C. auris*. First, in order to evaluate acute systemic toxicity of the postbiotic elements, the larvae were injected with different doses of LPCE and LPF1, and none of the systemic doses (10–80 mg/kg) resulted in death of the larvae. Additionally, we found that the injection of LPCE or LPF1 into *G. mellonella* larvae infected by *C. auris* increased the survival of these insects (43% for LPF1; 37% for LPCE), the number of hemocytes and the gene expression of galiomicin. These data further confirm the excellent performance of LPCE and LPF1, thus providing important insight into combating *C. auris*. To the best of our knowledge, this is the first article in the literature that uses postbiotic elements in *G. mellonella*.

Taken together, these findings indicate that LPCE and LPF1 are capable of stimulating the cellular and humoral immune responses of the larvae and consequently reduce *C. auris* infection. The use of posbiotics elements with antifungal and immunomodulatory properties could be promising in *C. auris* infection, once a recent study questioned the effectiveness of the immune system against this species. It was demonstrated that human neutrophils do not properly recognize *C. auris* as they do with other *Candida* species and this behavior may explain the high mortality rates even for patients treated with antifungals (Nett, 2019).

Modulation of the *G. mellonella* immune response has been reported by different studies using probiotics (Ribeiro et al., 2017; Rossoni et al., 2017; Scalfaro et al., 2017; Geraldo et al., 2019). The main cells involved in the cellular immune response of *G. mellonella* are hemocytes (Bergin et al., 2003; Sheehan and Kavanagh, 2018). These are responsible for important events such as nodulation, encapsulation, and phagocytosis. Phagocytosis is a very important cellular process in which some enzymes are released by hemocytes in order to destroy the invading pathogen (Kavanagh and Reeves, 2004; Pereira et al., 2018; Sheehan and Kavanagh, 2018). The humoral response of these insects is constituted by effector molecules including opsonins, melanin, and antimicrobial peptides (AMPs) (Tsai et al., 2016). Although there are 18 different types of AMPs identified in *G. mellonella* hemolymph, we chose to examine galiomicin because it is a specific *G. mellonella* defense and is one of the most effective AMPs against fungal infection (Wojda, 2017). This AMP shows homology to human cysteine-rich peptides from the ß group of defensins and make up part of the innate immune system. In general, AMPs are the last line of defense and they act on the hemolymph attacking elements of the bacterial or fungal cell wall (Shai, 2002; Mowlds et al., 2010; Rossoni et al., 2017).

Although postbiotic elements of *L. paracasei* 28.4 have shown promising results, the key components of LPCE and LPF1 with antifungal properties are still unknown and need to be further investigated. Future studies should be addressed for the isolation and characterization of the bioactive molecules presents in *L. paracasei* 28.4 supernatant, as well as for the action mechanisms of these postbiotic elements on specific targets in fungal cells.

Within the limitations of the study, it can be concluded that *L. paracasei* 28.4 cells and its postbiotic elements (LPCE and LPF1) have antifungal activity against *C. auris* including activity against planktonic and persister cells, as well as biofilms. Postbiotic derived from *L. paracasei* 28.4 protected *G. mellonella* infected with *C. auris* and enhanced its immune status indicating a dual function in modulating the host immune response. The exact mechanisms related to the action of postbiotic elements against *C. auris* are still unclear and need to be further investigated.

## Data Availability Statement

The datasets generated for this study are available on request to the corresponding author.

## Author Contributions

RR and PB conducted most of the experiments. DS, IM, and RM played a key role in extraction and fractionation of post-biotic elements. RR, BF, JJ, and EM wrote the paper. EM, BF, and JJ conceived and supervised all the work. All authors contributed to the article and approved the submitted version.

## Conflict of Interest

The authors declare that the research was conducted in the absence of any commercial or financial relationships that could be construed as a potential conflict of interest.

## Abbreviations

BHI: Brain heart infusion
CDC: Centers for Disease Control and Prevention
CFU: colony-forming unit
CLSI: Clinical and Laboratory Standards Institute
LPCE: *L. paracasei* crude extract
LPF1: *L. paracasei* fraction 1
MIC: minimum inhibitory concentration
MRS: Man, Rogosa and Sharpe
PBS: phosphate buffered saline
YNB: Yeast nitrogen base
YPD: Yeast peptone dextrose.
